# Multiple Damage Detection in PZT Sensor Using Dual Point Contact Method

**DOI:** 10.3390/s22239161

**Published:** 2022-11-25

**Authors:** Sayantani Bhattacharya, Nitin Yadav, Azeem Ahmad, Frank Melandsø, Anowarul Habib

**Affiliations:** 1 Department of Mining Machinery Engineering, Indian Institute of Technology Dhanbad, Dhanbad 826004, India; 2 Department of Electrical Engineering, Indian Institute of Technology Delhi, New Delhi 110016, India; 3 Department of Physics and Technology, UiT-The Arctic University of Norway, 9019 Tromsø, Norway

**Keywords:** Lead Zirconate Titanate, micro-defects, structural health monitoring, Haar Discrete Wavelet Transformation, point contact excitation and detection method

## Abstract

Lead Zirconate Titanate (PZT) is used to make ultrasound transducers, sensors, and actuators due to its large piezoelectric coefficient. Several micro-defects develop in the PZT sensor due to delamination, corrosion, huge temperature fluctuation, etc., causing a decline in its performance. It is thus necessary to identify, locate, and quantify the defects. Non-Destructive Structural Health Monitoring (SHM) is the most optimal and economical evaluation method. Traditional ultrasound SHM techniques have a huge impedance mismatch between air and solid material, and most of the popular signal processing methods define time series signals in only one domain, which provides sub-optimal results for non-stationary signals. Thus, to improve the accuracy of detection, the point contact excitation and detection method is implemented to determine the interaction of ultrasonic waves with micro-scale defects in the PZT. The signal generated from this method being non-stationary in nature, it requires signal processing with changeable resolutions at different times and frequencies. The Haar Discrete Wavelet Transformation (DWT) is applied to the time series data obtained from the coulomb coupling setup. Using the above process, defects up to 100 μm in diameter could be successfully distinguished.

## 1. Introduction

Lead zirconate titanate (Zr_x_Ti_1−x_)O_3_ (PZT) is a piezoelectric material with a large piezoelectric coefficient. PZT ceramics have negligible mass, easy and fast integration, large frequency responses, low power consumption, low cost of the sensors, superior electromechanical coupling, and impedance matching with various substrates [1,2,3]. These properties make PZT ceramics suitable for integration into the host structure as an in situ generator/sensor, and they are thus used extensively. 

In harsh and extreme environmental conditions, such as corrosion, fatigue, and delamination due to extreme temperature fluctuations, several surfaces and sub-surfaces are likely to be introduced with micro-defects within the sensor. These flaws may also be intrinsic to the bulk material, as seldom are they introduced in the final stages of the fabrication or early stages of device operation. Therefore, it is necessary to identify, locate, and quantify the defects in sensors to avoid structural failure and false alarm in structural health monitoring (SHM) applications

In the past several years, a wide range of innovative methods has been implemented for Non-Destructive Evaluation (NDE) techniques for detecting intrinsic and bulk damage of ceramic components [4,5,6,7,8]. The air-coupled ultrasonic techniques are being increasingly used for material characterization, non-destructive evaluation of composite materials using guided waves, as well as distance measurements [9,10]. The main drawback of air-coupled ultrasound is the huge impedance mismatch between air and any solid material. Apart from ultrasonic methods, several optical methods have been considered to identify the surface and subsurface flaws in PZT ceramics. The most common optical measurements include photoacoustic microscopy, optical coherence tomography, and the optical gating technique [11,12,13]. The scanning laser Doppler vibrometer (SLDV) has been employed for three-dimensional visualizations of acoustic wave interference with inclusions and flaws in metallic structures, piezoelectric crystals, and piezo-ceramics [14,15]. Other, notable signal processing techniques that are widely accepted in SHM applications are Singular Spectrum Analysis, Frequency Domain Decomposition (FDD), the Auto-Regressive Model, and Extended Kalman Filter Weighted Global Iteration techniques [16,17,18,19,20,21]. These techniques provide a damage index parameter based on the spectral content or statistical evaluation of the time series. Generally, processing the stationary signal is easier as, with the help of the entire dataset, the statistics of a signal can be evaluated, and this information can be used to derive a conclusion. In contrast, for non-stationary signals to derive any information, algorithms must be adapted for use. Bordeaux and Golinval [22], Sohn et al. [23,24], and Yao and Pakzad [25] have developed an adaptive feature extraction approach. These approaches assume the behavior of the structure to be linear and detect the damage with help of changes in extracted features or the proposed novel damage index derived from these features [26].

In the last several years, a significant amount of effort has been devoted to improving the point contact excitation and detection method to excite the acoustic waves in piezoelectric crystals and ceramics [27,28,29,30,31,32,33]. The point contact excitation and detection method is a unique way to generate acoustic waves in piezoelectric materials in the absence of coupling media; mechanical, geometrical, and electrical resonances; and photolithography [34]. The working principle of this technique depends on the transfer of the electromagnetic field to mechanical energy to excite phonon vibration in piezoelectric materials [28]. The Coulomb coupling method and spectral decomposition technique have been implemented for the localization of surface defects in piezo-ceramic structures wherein the signal processing is performed using Fast Fourier Transform. Unlike Fast Fourier Transform, wavelet transformation can define any type of signal in both the time and frequency domains simultaneously and has fast computation.

## 2. Experimental Setup

Our group has previously provided a complete overview of the experimental setup for the point contact excitation and detection method [27,28,29,30,31,32,33,34,35]. The point contact excitation and detection scheme is based on the Coulomb coupling phenomenon [36]. This phenomenon works on the transfer of electric energy to mechanical energy and vice versa. In this study, a high voltage was applied to the sender probe (steel sphere) that acts as a Coulomb source. In Coulomb coupling, the radius of the sender probe plays a vital role [28]. The spatial derivatives of the electric field are not zero on the crystal’s surface or inside the sample when the conductive sphere is considered to be an electrical point contact source. Thus, we can assume that the surface terms dominate the coupling of the electric field to the strain gradient under the first approximation. Therefore, the point contact excitation and detection method is well suited for the detection of surface anomalies and surface defects/cracks. The complete experimental setup consists of four basic processes, i.e., (1) probe fabrication for both sending and receiving electrodes, (2) PZT sample preparation, (3) damage insertion on the PZT sample, and (4) data acquisition.

The sender probe, which consists of a steel sphere at the point of a triangle made of two fiber optic cables and a printed circuit board (PCB), is carefully pressed against the PZT ceramic surface. This probe was used as a Coulomb exciter for the generation of ultrasonic waves in the piezo-ceramics. For the purpose of receiving the transmitted waves, a second similar probe is positioned on the opposing side of the sample. For the continuous excitation and detection of acoustic waves in PZT ceramics, these dual probes work together.

**Figure 1:**
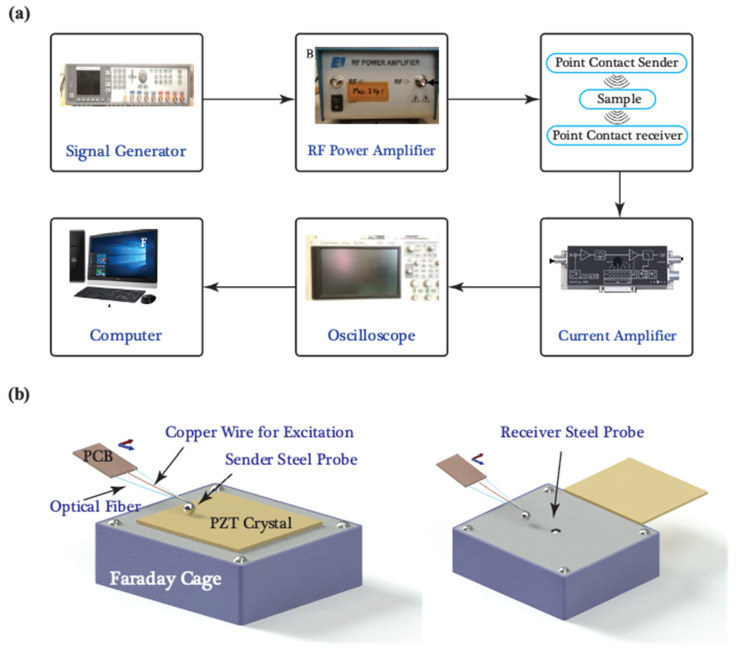
(**a**) Experimental setup for point contact excitation and detection scheme and (**b**) 3D illustration of the arrangement of sender and receiver steel probe along with the PZT sample.

**Figure 2:**
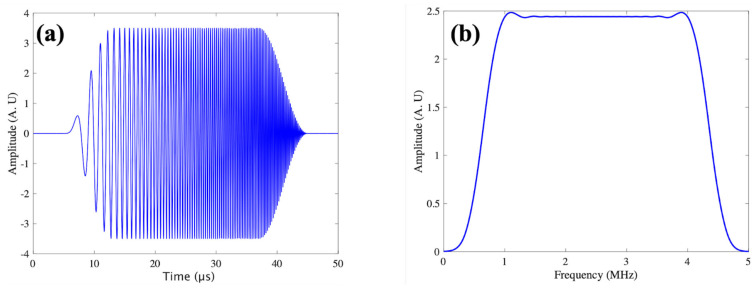
The excitation signal (Chirp) used in the experiment, the excitation signal (**a**), and its Fourier spectrum (**b**).

Figure 1 represents the experimental setup used in this paper. In Figure 1a, a signal generator of arbitrary functions (Agilent 81150A) delivered a chirp-coded signal of width 45 μs (sampling frequency 200 MHz) to a radio frequency (RF) amplifier (Electronics & Innovation: 403LA, New York, NY, USA) for signal amplification. These amplified signals were then delivered to the steel sphere for the excitation of acoustic waves in the PZT sample. Figure 1b presents a 3D illustration of the sender and receiver steel probe along with the PZT sample. On the opposite side of the PZT, an identical probe was used to acquire the propagated signal. The received signals were fed into a trans-impedance amplifier (DHPCA-100) for signal amplification. Finally, the amplified signal was acquired using an oscilloscope (Agilent 3024A) capable of digitizing with up to 12 bits. The oscilloscope averages 256 pulse shots and digitizes the received signal, which is then saved in a personal computer (PC) via a USB port. The PC also controls the mechanical scanner in the XY plane, i.e., the step size is 50 µm in both directions with a scanning area of 10 mm × 10 mm. The excitation signal (a) and its Fourier spectrum (b) are shown in Figure 2.

## 3. Methodology

The major challenge in structural health monitoring is the detection of micro-damages in the solid structure. There are various techniques mentioned above by which one can detect the damages, i.e., a combination of various signal processing and analytical techniques. This paper focuses on the point contact excitation and detection method along with two different signal processing techniques for damage detection: (i) analysis of the power spectral density of the signals, and (ii) analyzing coefficients by Haar (db1) discrete wavelet transformation (DWT) of the signals.

### 3.1. Power Spectral Density Analysis of the Signal

Fourier Transform decomposes time series data into a sum of infinite sine and cosine functions of different amplitudes and frequencies. This process converts a waveform that is difficult to describe mathematically into a more manageable series of trigonometric functions which, added together, reproduces the original waveform. Fourier Transforms are of two types: Discrete and Continuous, where, when distinct ordered pairs representing the original input function are equally spaced in their input variable (equal time steps, in this case), this is called Discrete Fourier transform (DFT), while ordered pairs with input variables with an infinitesimal difference between them is called Continuous Fourier Transform.

Fourier Transform (F) of a function *f*(*t*) is given by the following expression:

(1) $$F\left(w\right)=\int_{-\infty }^{\infty }f\left(t\right)e^{-2\pi itw}dt$$

where *t* is time and *w* is the frequency in Hertz.

Discrete Fourier Transform (S) of a function *f*(*x*) is given by the following expression:

(2) $$S\left(w\right)=\sum_{i=0}^{N-1}e^{-jw\left(i△t\right)}\;y_{i}\;(i\Delta t)\Delta t$$

where *N* is the total no. of equally spaced data points and *y_i_*(*i*Δ*t*) is the actual data recorded at i*_th_* time.

Many time series functions show complicated periodic behavior. Spectral analysis is a technique that helps in discerning these underlying periodicities. To perform spectral analysis, data are first transformed using Fast Fourier Transform (FFT) from the time domain to the frequency domain. FFT is a computationally efficient algorithm for solving DFT faster by reducing the number of redundant calculations. Power Spectral Density of a signal analyses the distribution of power as a function of frequency over the entire frequency range.

The mathematical representation of PSD is:

(3) $$P_{\mathrm{ab}}=\frac{1}{2\pi }\int_{wa}^{wb}S\left(w\right)\;dw$$

### 3.2. De-Noising by Wavelet Transformation (DWT) of the Signal

**Chart 1:**
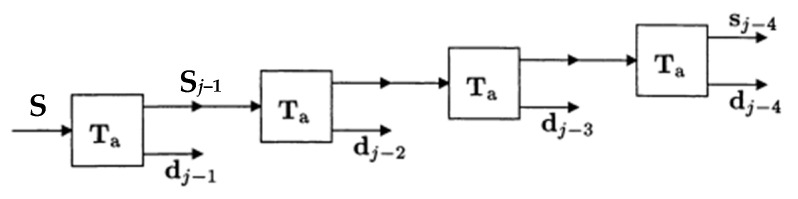
The above flowchart represents DWT over 4 scales, wherein *j* is the number of times the transformation algorithm is applied to the original signal $\left(S_{j}\right)$.

In Method 1, normalized FFT is used. Unlike FFT, Wavelet Transform (WT) can extract information from both spectral and temporal regions, thus ensuring more resolute signal processing. Wavelet Transform decomposes a signal into multiple lower-resolution levels by varying the scaling and shifting factors of a single wavelet function (mother wavelet). In the first step, the time series is decomposed into two high and low frequency components. Then, high frequencies are retained, while low frequencies are decomposed again into two high and low frequencies. High frequencies are called the details coefficient and low frequencies are the approximation coefficient of the signal. Wavelet Transforms are of two types, Continuous Wavelet Transform (CWT) and Discrete Wavelet Transform (DWT). CWT is very slow due to the extra data that overlap with its neighboring data (duplicity). Therefore, DWT is used in this paper. This paper uses the first member of the Daubechies family of orthogonal discrete wavelets, popularly known as the Haar Wavelet. One of the main reasons behind choosing the Haar wavelet is that it is computationally fast and memory efficient, as it can be calculated in place without the need for temporary array allocation. A Haar wavelet is a discontinuous step function. These abrupt changes in the function are beneficial for the analysis of signals with sudden transitions. The half-band high-pass (G) and half-band low-pass (H) filters are given by G=[12 12] and H=[12 12] . Chart 1 represents DWT over 4 scales.

$$\mathrm{Mother}\;\mathrm{Wavelet}\;\mathrm{of}\;\mathrm{Haar}\;\left((n)\right)=\{\begin{array}{c} 1\;0\leq n\leq 1/2\\ -1\;\frac{1}{2}\leq n\leq 1\\ 0\;otherwise \end{array}$$

where:

$S_{j}$—Original input signal,

$S_{j-1}$—Approximation coefficient at level 2,

$d_{j-1}$—Details coefficient at level 2,

$T_{a}$—Direct transform.

T_a_ is the building block of DWT. The formulae used in a T_a_ block of Haar Transform are:

1. $d_{j-1}\left[n\right]=S_{j}\left[2n+1\right]-S_{j}\left[2n\right]$,
2. $S_{j-1}\left[n\right]=S_{j}\left[2n\right]-\frac{1}{2}\times d_{j-1}\left[n\right]$,
3. $S_{j-1}\left[n\right]=\sqrt{2S_{j-1}\left[n\right]}$,
4. dj−1[n]=12dj−1[n].

## 4. Procedure

### 4.1. Power Spectral Analysis on Received Signal

**Chart 2:**
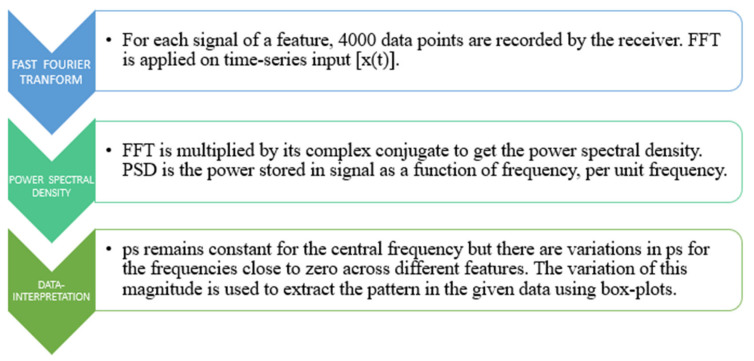
The flowchart explains the three steps (i.e., Fast Fourier Transformation, power spectral density calculation, and data interpretation) performed to obtain the box plots from the experimental data.

**Figure 3:**
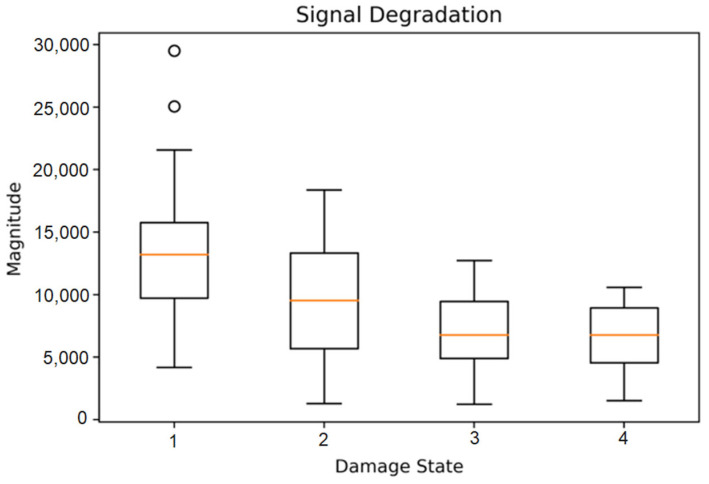
The figure consists of four box plots, one of each damage state (damage size of 500 µm). The individual box plot computes the variation in the second maxima amplitude value of PSD plots. As explained in Section 4.2, the plot approximately represents the power stored by those signals.

A steel probe receives the signal in the form of time series data. For the same damage state and size, 12 different datasets are measured from uniformly distributed axis points. To extract meaningful information, power spectral (PS) analysis is performed. PS value is calculated for each time series of 4000 data points, and 12 such values were obtained. This was then converted into a box plot for that damage state as mentioned in Chart 2. Boxplots of four damage states are then compared in Figure 3.

### 4.2. Haar Wavelet Analysis on Received Signal

**Chart 3:**
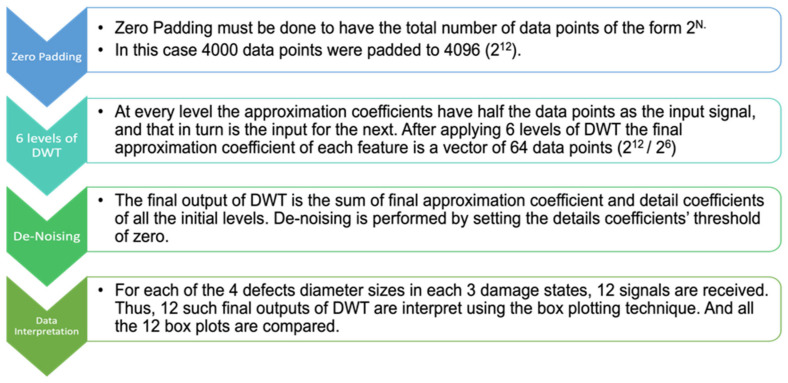
The above flowchart explains the four steps (i.e., Zero-Padding, DWT Calculation, de-noising, and data interpretation) performed to obtain the box plots from the experimental data.

A steel probe receives the signal in the form of time series data. For the same damage state and size, 12 different datasets are measured from uniformly distributed axis points. To extract meaningful information from the data, de-noising is performed using Haar discrete wavelet analysis. The final output is the summation of detailed and approximation coefficients of all six levels (explained in Chart 1 and Chart 3). Approximation coefficients are equated to zero, and the average of the values thus obtained are considered 12 times to develop the box plot of that damage size and damage state. For each damage state, four box plots of different sizes are compared, and, in total, four such damage states are used in this experiment.

## 5. Experimental Observation

### 5.1. Power Spectral Density Analysis

It can be observed from Figure 3 that with the increase in the damage state, the statistical median of power reduces. Observations of the figures shown above are as per the expectations because, with the increase in damage in the sample, the signal will be interrupted to a greater extent, leading to a loss of information and reduction in power. A similar trend is observed for damage sizes of 600 μm, 800 μm, and 900 μm. This variation in median values is distinct enough to distinguish between the different damage states. Thus, it can be concluded that the PSD analysis method can be effectively used for understanding the degree of damage in PZT ceramics.

### 5.2. Discrete Wavelet Transformation

**Figure 4:**
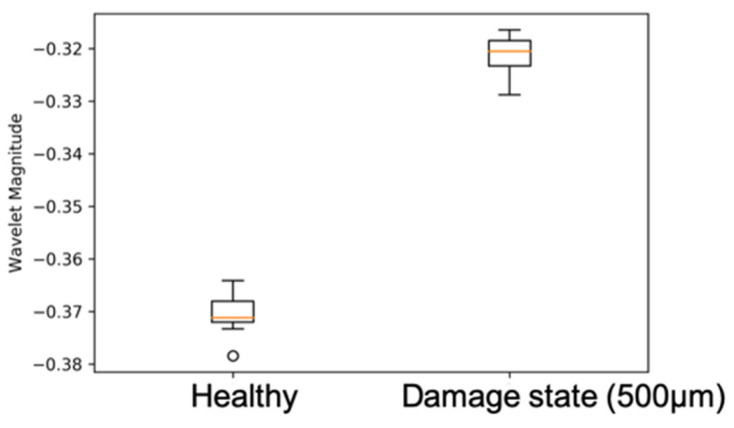
The above box plots are of the de-noised signal, as described in the procedure. The plot on the left is of a healthy PZT sample, and the one on the right is of a PZT ceramic sample with 500 μm damage size in the 1st damage state.

Figure 4 represents the health and damage state for the 500 μm damage size. Here, it is represented as damage state 1.

**Figure 5:**
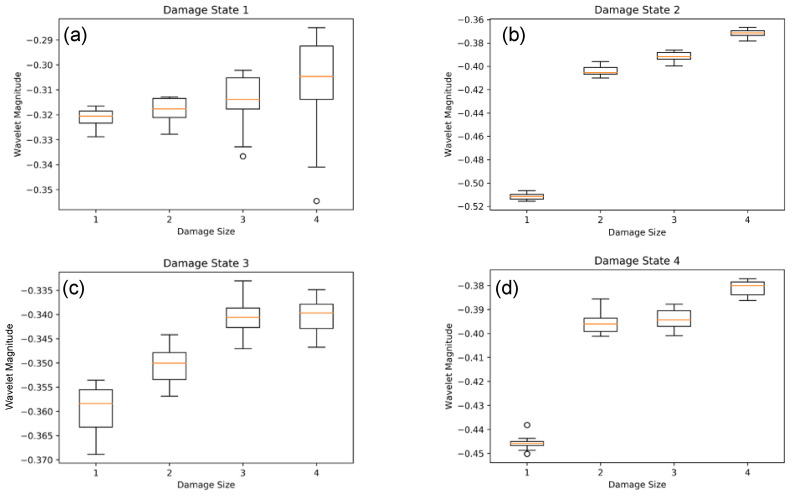
Damage sizes 1, 2, 3, and 4 correspond to damage sizes 500, 600, 800, and 900 μm, respectively. Each of the subplots in the figure (**a**–**d**) shows the comparison between the different damage sizes of a particular damage state. The boxplot of all four damage states is shown above, in ascending order of progression.

Figure 5 represents the different damage sizes for different damage states. Here, damage sizes 1, 2, 3, and 4 correspond to damage sizes 500, 600, 800, and 900 μm, respectively.

Figure 5a shows damage state 1, which includes four different damage sizes. Figure 5b–d represents damage states 2, 3, and 4, respectively. A clear distinction can be observed in the statistical medians of the box plots in Figure 4 and Figure 5. Figure 4 proves that the Haar DWT de-noising method is efficient for damage detection. Figure 5 is used as a comparison chart between all the 12 damage states. Damage of multiple sizes (500, 600, 800, and 900 μm) and multiple locations (damage states 1, 2, 3, and 4) is represented together, and the observable distinction in their amplitude values can prove that this method is successful in distinguishing between all of them.

It can also be concluded from Figure 4 that DWT is more efficient compared to PSD analysis. PSD was successful in distinguishing between the damage states of the PZT sample, while DWT can also distinguish between different damage sizes (with 100 μm variation in dimension) within the same damage state.

## 6. Conclusions

This paper targets a common but challenging problem of damage detection and damage classification for small differences in damage size. The point contact excitation and detection method is used in this experiment to extract raw data. As the signals are very sensitive to noises, it was challenging to interpret the raw data. Therefore, a couple of signal processing techniques (i.e., power spectral analysis and wavelet transformation) are used to extract meaningful information from the main signal. In power spectral analysis, the phenomenon that structural damage leads to power reduction is used to discern different damage states. The discrete wavelet transformation with greater resolution is used to distinguish between the damage states as well as between damage sizes up to a difference of only 100 μm for the same damage state. Thus, according to the results, the proposed methods were successful in distinguishing PZT ceramic samples based on structural damage severity. The experimental method (point contact) presented here is limited to only piezoelectric materials. A deposited thin layer of piezoelectric film (AlN or ZnO) on the sample surface would be a good option to overcome such a problem. In such an experimental condition, the point contact method can open up a new avenue in the field of SHM of multi-defects detection and localization of structures.

## Data Availability

Data will be available upon request.
